# Gut microbiota and risk of coronary heart disease: a two-sample Mendelian randomization study

**DOI:** 10.3389/fcvm.2024.1273666

**Published:** 2024-03-25

**Authors:** Xiang-zhi Hu, Ling-ling Fu, Bin Ye, Man Ao, Ming Yan, Hong-chao Feng

**Affiliations:** ^1^ Medical College, Guizhou University, Guiyang, China; ^2^ Department of Oral and Maxillofacial Surgery, Guiyang Hospital of Stomatology, Guiyang, China; ^3^ Department of Oral and Maxillofacial Surgery, University Medical Center Hamburg-Eppendorf, Hamburg, Germany

**Keywords:** coronary heart disease, gut microbiota, causal inference, Mendelian randomization study, inverse variance-weighted

## Abstract

**Background:**

The relationship between gut microbiota composition and coronary heart disease (CHD) has been recently reported in several observational studies. However, the causal effect of gut microbiota on coronary heart disease is uncharted.

**Objective:**

This study attempted to investigate the effect of gut microbiota on coronary heart disease by Mendelian randomization (MR) analysis.

**Methods:**

Through the two-sample MR method, single-nucleotide polymorphisms relevant to gut microbiota were selected as instrument variables to evaluate the causal association between gut microbiota and the risk of CHD.

**Results:**

According to the selection criteria of the inverse variance-weighted average method, Class Actinobacteria, Class Lentisphaeria, Family Clostridiales vadinBB60group, Genus *Clostridium innocuum* group, Genus *Bifidobacterium*, Genus *Butyricicoccus*, Genus *Oxalobacter*, Genus *Turicibacter*, and Order Victivallales, presented a suggestive association with coronary heart disease.

**Conclusion:**

This two-sample Mendelian randomization study found that gut microbiota was causally associated with coronary heart disease. Further randomized controlled trials are needed to clarify the protective effect of probiotics on coronary heart disease and their specific protective mechanisms.

## 1 Introduction

The gut bacteria present in human intestines are a large population of bacteria that constitute the largest microbiota in the body (1). The human intestine contains at least 1,000 species of bacteria, with a total of more than 100 million bacteria, forming a complex group (2). The genes encoding these microbes are at least one billion times larger than the human genome.

Many scholars have done many profound studies on the physiological processes of gut bacteria in humans, and since the discovery of gut flora (3), it has been acknowledged that the gut bacteria flora play an important role in regulating the nervous system and metabolism and immunity, and a delicate balance is maintained between them and humans (4).

Coronary heart disease (CHD) is damage to the myocardium caused by an imbalance between the blood supply to the coronary arteries and the demand on the myocardium due to functional or organic pathology, also known as ischemic heart disease (5). The most common cause of coronary heart disease is atherosclerosis, which accounts for about 90% of cases and is a chronic, progressive inflammatory disease that occurs in the vascular system. Atheromatous plaques in the coronary arteries gradually increase in size and cause. The main mechanism of coronary heart disease is the blockage of blood flow or the exposure of endothelial collagen fibers as a result of plaque rupture, leading to thrombosis. This is regulated by a combination of inflammatory factors and metabolic substances (6).

In healthy people, the gut microbiome is made up mostly of good bacteria, with few bad ones. These two bacterial types are in homeostasis to maintain a healthy state of the host (7). Previous studies have shown that imbalances in the gut microbiome are strongly associated with infectious and inflammatory and metabolic diseases (8). The imbalance of gut microbiota leads to the disorder of bacterial structure and the destruction of basic metabolic processes of the host, which may be closely related to the occurrence of cardiovascular diseases such as coronary heart disease, hypertension, and heart failure (9).

Studies have shown that gut bacteria are associated with many other risk factors for coronary heart disease, such as obesity, diabetes, high blood cholesterol, and high blood pressure (10). The gut microbiome has been observed to play an important role in coronary heart disease. Karlsson (11) found that the number of *Collinsella* bacteria increased in patients with coronary heart disease compared with healthy people, while the count of *Rothia* and *Eubacterium* spp. bacteria decreased. Emoto et al. (12) found that a significant increase in the number of mature lactic acid bacteria and a significant decrease in the number of bacteriophages (*Bifidobacterium* and *Prevotella*) were found in patients with coronary artery disease (13). Furthermore, there was a significant increase in the proportion of the thick-walled phylum/bacteroidetes (14). The proportion of lactobacilli in the gut microbiota of patients with coronary artery disease who did not use antibiotics was significantly higher and the proportion of *Bacillus mimicus* was significantly lower (15).

**Figure 1:**
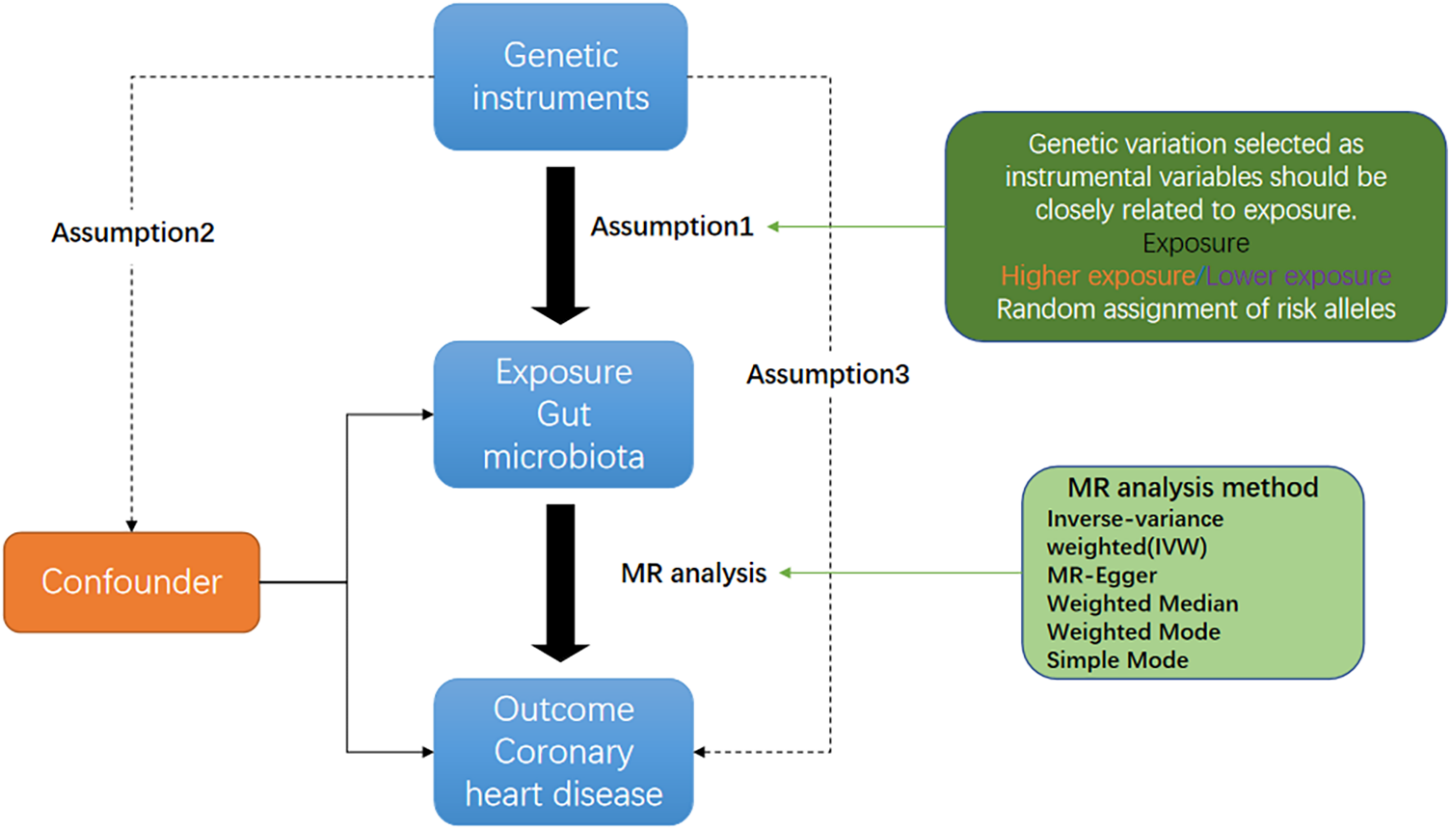
Three corresponding principal assumptions in this two-sample Mendelian randomization study.

Figure 1 shows an overview flow diagram of the Mendelian randomization (MR) hypothesis. This study used MR to explore the causal association between gut microbiota and coronary heart disease (16). In MR, the causal relationship between exposure and disease outcomes is estimated through instrumental variables (IV) used to construct genotypes (17). The random distribution of genotypes is designed according to Mendelian laws of inheritance. If the genotype determines the phenotype, then the genotype is associated with the disease through the phenotype, so the genotype can be used as an instrumental variable to infer the association between the phenotype and the disease. It uses genetic variants strongly associated with exposure factors as instrumental variables to assess the causal relationship between exposure factors and outcomes. There are three hypotheses of Mendelian randomization: (1) Association hypothesis: single-nucleotide polymorphisms (SNPs) are strongly correlated with exposure factors; (2) Independence hypothesis: SNPs are independent from confounders; (3) Exclusivity hypothesis: SNPs can only have an effect on outcomes through exposure factors. For studying the causal relationship between gut microbiota and diseases (including metabolic diseases), MR has been widely used for studying autoimmune diseases and rheumatoid arthritis (18). A genome-wide association study (GWAS) summary from the MiBioGen and MR-Base consortiums was used in this study, and coronary heart disease and gut microbiota were evaluated using MR analysis of two samples (19).

## 2 Methods

### 2.1 Data sources

The MiBioGen consortium conducted the largest genome-wide meta-analysis to date for gut microbiota composition to identify genetic variants. The study included 18,340 individuals from 24 cohorts from the USA, Canada, Israel, Germany, Denmark, the Netherlands, Belgium, Sweden, Finland, and the UK (20). To identify host genetic variants associated with the abundance levels of bacterial taxa, a microbiota quantitative trait loci mapping analysis was performed. The study identified 131 genera with a mean abundance greater than 1%, including 12 genera that were unknown. Therefore, 119 genus-level taxa were included in the current study for analysis. CHD in this study was defined as heart disease involving plaque buildup in the heart arteries (atherosclerosis) and reduced blood flow to the heart muscle, resulting in myocardial ischemia, hypoxia, or necrosis. Summary statistics for CHD were developed using the GWAS summary dataset for CHD obtained from the IEU Open GWAS project (ieu-a-7) (21). A total of 184,305 adult subjects and 123,504 controls participated in this GWAS. Sex, age, first 10 principal components, and genotyping batch were corrected during the analysis (22).

### 2.2 Instrumental variable

The screening of IVs in MR studies is consistent with the aforementioned three core hypotheses of MR: (1) Association hypothesis: SNPs are strongly correlated with exposure factors, with *F*-value > 10 as the closely related criterion; (2) Independence hypothesis: SNPs are independent from confounders. (3) Exclusivity hypothesis: SNPs can only have an effect on outcomes through exposure factors. IVs were selected based on the following criteria: (1) Statistically significant SNPs in each genus (*P* < 1.0 × 10^–5^) are considered potential IVs; (2) A reference panel of European samples was used to calculate linkage disequilibrium (LD) between SNPs in the 1000 Genomes project, and among those SNPs that had *R*^2^ < 0.001 (clumping window size = 10,000 kb), only the SNPs with the lowest *P*-values were retained; (3) SNPs with minor allele frequency (MAF) ≤0.01 were removed; and (4) Allele frequency information was used to determine forward strand alleles when palindromic SNPs existed (23). Basic information on the instrumental variables is in the Supplementary material S1 (Basic information on instrumental variables).

### 2.3 Statistical analysis

To verify whether there was a correlation between exposure to gut microbiota and the outcome CHD, MR analysis was conducted using five methods such as the inverse variance-weighted average method (IVW) (23), the weighted median method (24). MR-Egger regression analysis (25), and weighted mode. In addition, Cochran's IVW Q was utilized to quantify the heterogeneity of IVs (26). Further, to identify potentially heterogeneous SNPs, a “leave-one-out” analysis was performed by ignoring each tool for analyzing SNPs in turn (27).

All statistical analyses were performed using R version 4.2.1. MR analyses were performed using the TwosampleMR (version 0.5.6), MR-PRESSO (version 1.0), and *q*-value R packages (28).

## 3 Results

### 3.1 SNP selection

According to the IVs screening criteria, 128, 235, 294, 516, 1761SNPs associated with the intestinal microbiome were identified at the phylum, class, order, family, and genus levels. After a series of quality control, a total of 2,906 IVs were determined.

The *F* statistics of IVs were all >10, indicating that there was no evidence of weak instrument bias. According to the third hypothesis of Mendelian randomization, IVs must pass the exposure to affect the outcome. If the IVs can directly affect the result without exposure, then the idea of Mendelian randomization is violated, that is, the test results have horizontal pleiotropy. Therefore, the main premise of causality inference using Mendelian randomization is that there is no horizontal pleiotropy. We used the MR-PRESSO method to examine horizontal pleiotropy (MR-PRESSO global test *p* > 0.05; Supplementary File: res_presso).

Bacterial genera containing multiple SNPs were tested using four MR methods to account for multiple test corrections in the set of SNPs used as IV that are smaller than the genome-wide statistical significance threshold (1 × 10^−5^). According to the selection criteria for IVs, a total of 2,458 SNPs were used as IVs for 211 bacterial genera.

For all instrumental variables, their *F* statistics are all greater than 10, and all weak instrumental variables were excluded (Supplementary File: res_data).

As presented in Table 1, nine bacterial genera were found to be associated with CHD in IVW. Among them, Class Actinobacteria, Genus *Clostridium innocuum* group, Genus *Bifidobacterium*, Genus *Oxalobacter*, and Genus *Turicibacter*, show by analysis of the results that they are related to coronary heart disease (OR > 1); however, MR analysis results of Class Lentisphaeria, Family Clostridiales vadinBB60 group, Genus *Butyricicoccus*, and Order Victivallales reflect potential protection from CHD.

**Table 1:** Causal effects of gut microbiota on CHD.

Bacterial taxa (exposure)	Number of SNPs	MR method	*P*-value	OR	95% CI
Class Actinobacteria	22	MR-Egger	0.91	0.99	0.82–1.19
		Weighted median	0.02	1.12	1.02–1.24
		Inverse variance-weighted	0.04	1.08	1.00–1.16
		Simple mode	0.11	1.17	0.97–1.41
		Weighted mode	0.07	1.16	0.99–1.35
Class Lentisphaeria	8	MR-Egger	0.30	0.87	0.69–1.11
		Weighted median	0.18	0.94	0.86–1.03
		Inverse variance-weighted	0.02	0.92	0.87–0.99
		Simple mode	0.45	0.94	0.83–1.08
		Weighted mode	0.53	0.95	0.83–1.10
Family Clostridiales vadinBB60 group	15	MR-Egger	0.18	0.86	0.71–1.06
		Weighted median	0.07	0.91	0.83–1.01
		Inverse variance-weighted	0.02	0.92	0.86–0.99
		Simple mode	0.28	0.91	0.78–1.07
		Weighted mode	0.21	0.90	0.78–1.05
Genus *Clostridium innocuum* group	9	MR-Egger	0.62	1.09	0.77–1.57
		Weighted median	0.53	1.02	0.94–1.12
		Inverse variance-weighted	0.02	1.07	1.01–1.15
		Simple mode	0.61	1.03	0.91–1.18
		Weighted mode	0.62	1.03	0.91–1.18
Genus *Bifidobacterium*	20	MR-Egger	0.41	1.09	0.88–1.37
		Weighted median	0.01	1.13	1.03–1.24
		Inverse variance-weighted	0.03	1.07	1.01–1.16
		Simple mode	0.23	1.11	0.94–1.32
		Weighted mode	0.09	1.12	0.99–1.28
Genus *Butyricicoccus*	8	MR-Egger	0.07	0.82	0.68–0.99
		Weighted median	0.06	0.87	0.75–1.01
		Inverse variance-weighted	0.01	0.87	0.79–0.97
		Simple mode	0.10	0.81	0.66–1.01
		Weighted mode	0.08	0.85	0.74–1.00
Genus *Oxalobacter*	11	MR-Egger	0.19	1.20	0.93–1.56
		Weighted median	0.02	1.08	1.01–1.17
		Inverse variance-weighted	0.01	1.08	1.03–1.15
		Simple mode	0.06	1.11	1.01–1.24
		Weighted mode	0.07	1.11	1.00–1.23
Genus *Turicibacter*	10	MR-Egger	0.82	1.04	0.73–1.50
		Weighted median	0.14	1.08	0.97–1.22
		Inverse variance-weighted	0.01	1.12	1.03–1.23
		Simple mode	0.62	1.05	0.86–1.29
		Weighted mode	0.56	1.05	0.88–1.26
Order Victivallales	8	MR-Egger	0.30	0.87	0.69–1.11
		Weighted median	0.16	0.94	0.86–1.03
		Inverse variance-weighted	0.02	0.92	0.87–0.99
		Simple mode	0.48	0.94	0.82–1.09
		Weighted mode	0.53	0.95	0.83–1.10

OR, odds ratio; CI, confidence interval.

Cochran's IVW *Q* test exhibited no heterogeneity in these IVs (Supplementary File: res_hete).

Potential outliers were present in the IV of *Clostridium innocuum* group, *Oxalobacter*, and *Turicibacter* in visual tests on scatter plots and retention plots. MR-PRESSO analysis further, however, did not find any significant outliers. Thus, there was not enough evidence to show a pleiotropic relationship between these bacteria and CHD level.

**Figure 2:**
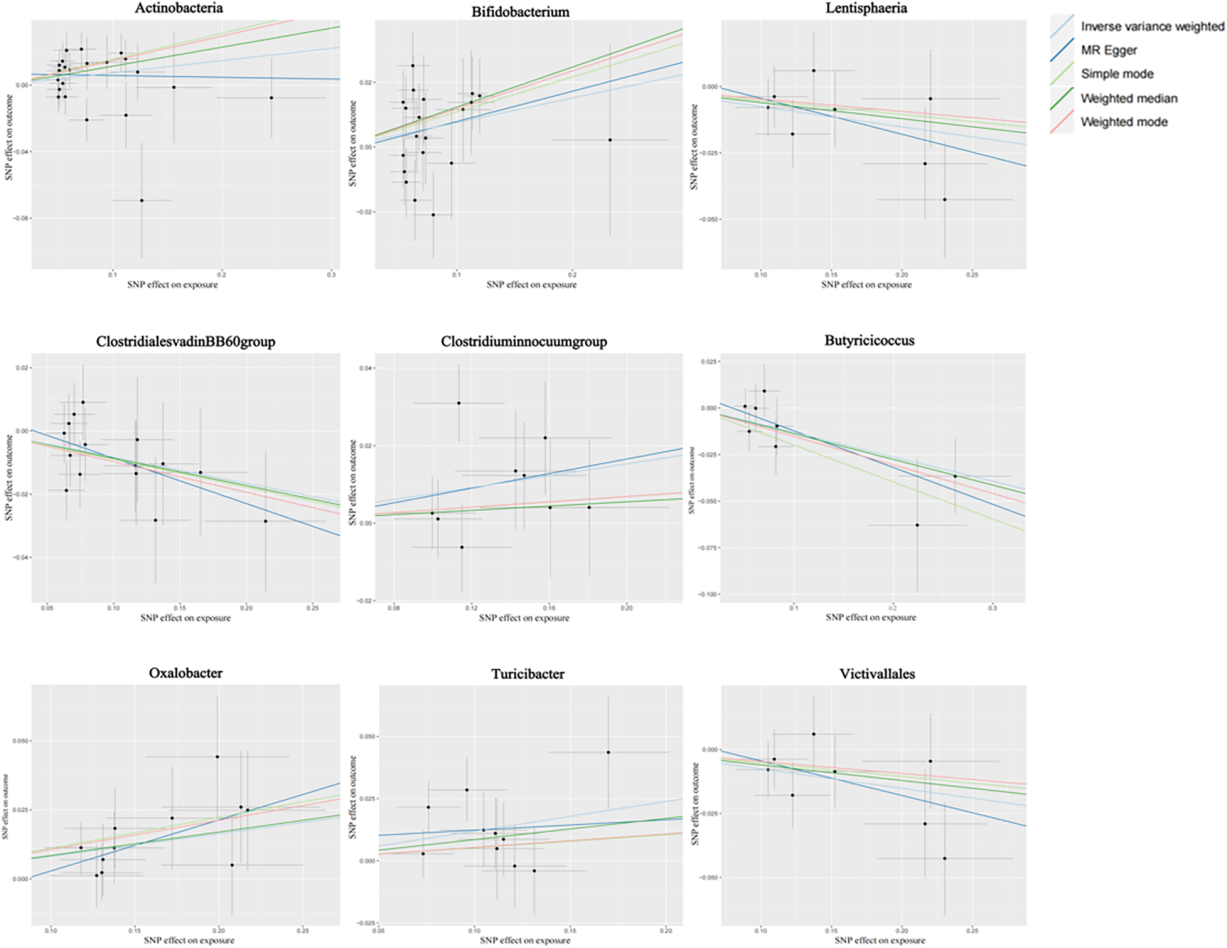
Causal effects of gut microbiota on CHD outcomes.

Figure 2 shows the causal association between gut microbiota and CHD. The correlation between gut microbiota and CHD was visualized in a scatter plot. In this plot, each black dot represented an SNP. With the correlation between SNP and exposure taken as the *X* axis, and the correlation between SNP and outcome taken as the *Y* axis, the slope of the drawn line marked the potential causal correlation of each method.

It was observed that Class Lentisphaeria, Family Clostridiales vadinBB60 group, Genus *Butyricicoccus*, and Order Victivallales could inhibit the occurrence of CHD, exerting a protective effect, while Class Actinobacteria, Genus *Clostridium innocuum* group, and Genus *Bifidobacterium*, Genus *Oxalobacter*, and Genus *Turicibacter* were positively correlated with the occurrence of CHD.

**Figure 3:**
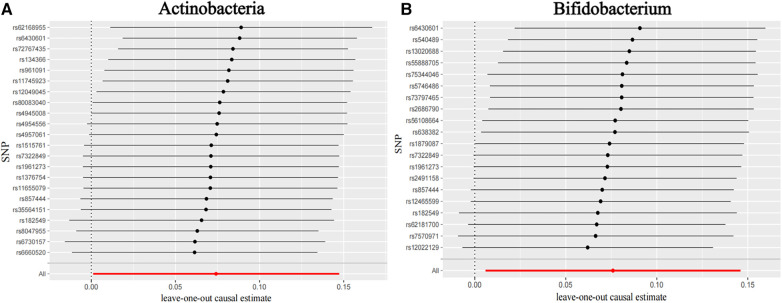
Leave-one-out plots for the causal association between gut microbiota and CHD. (**A**) Actinobacteria and (**B**) *Bifidobacterium*.

Figure 3 shows the causal association between Actinobacteria, *Bifidobacterium*, and CHD. The leave-one-out analysis showed no significant difference in the causal association between the aforementioned nine bacterial genera and CHD. After eliminating each SNP as an IV one by one, the overall trend did not change significantly, that is, no SNP was found with a great impact on the outcome among IVs. Visualizations of other bacteria are shown in the Supplementary material S2 (Visualizations of leave-one-out results).

## 4 Discussion

In this study, pooled statistics for gut microbiome from the largest GWAS meta-analysis conducted by the MiBioGen consortium and pooled statistics for coronary heart disease from the GWAS pooled dataset were used (29).

The gut microbiome is a complex and dynamic collection of ecological microbial communities colonized in the human gut. These bacteria play a vital role in the homeostasis of the digestive system and the health of the host, with a variety of metabolic, immune, and protective functions (30). System development and the function of gut bacteria stabilize and increase in diversity as age grows. Based on more than 450,000 European integrated genetic data (31), we have identified a number of genetic predispositions in the gut microbiome that are causally associated with CHD. We also identified that some may be a potential risk factor for CHD gut microbiota. These results may be designed to reduce the risk of coronary heart disease by public health interventions, hence they have enlightenment significance.

In this study, the GWAS summary statistics of intestinal bacteria from MiBioGen Alliance and the GWAS summary statistics of coronary heart disease from MR-Base were used to conduct MR analysis on the two samples to study the causal relationship between gut microbiota and CHD. In this study, five species of bacteria (Class Actinobacteria, Genus *Clostridium innocuum* group, Genus *Bifidobacterium*, Genus *Oxalobacter*, Genus *Turicibacter*) have shown potential role in CHD, and four kinds of bacteria (Class Lentisphaeria, Family Clostridiales vadinBB60 group, Genus *Butyricicoccus*, Order Victivallales) have shown protective effect on CHD.

Through its metabolites, the gut microbiome is involved in mediating basic metabolic processes, such as cholesterol metabolism, uric acid metabolism, oxidative stress, and inflammatory response, which can lead to atherosclerosis and coronary heart disease.

Many observational studies have reported a link between gut microbiota and coronary heart disease. *Bifidobacterium* is one of the most prominent probiotics, which is generally considered safe and friendly bacteria (32). However, in our study, it was positively associated with coronary heart disease, which we believe may be due to the following reasons. First, the GWAS data of gut microbiota we used remained at the genus level, and the specific data of *Bifidobacterium* species have not been counted, so detailed GWAS data of different species of *Bifidobacterium* are needed to conduct further MR research. Secondly, with the deepening of research on *Bifidobacterium*, the types of *Bifidobacterium* found are also gradually increasing. In the latest study, the *Bifidobacterium* obtained based on 16S rDNA sequencing technology was divided into 34 different species, including *Bifidobacterium adolescium*, *Bifidobacterium keratobium*, and *Bifidobacterium animalis* (33). The roles and benefits of some *Bifidobacterium* species have been found, including immune regulation, promoting tolerance, and playing a protective role in cardiovascular diseases. However, the effects and mechanisms of most *Bifidobacterium* species on CHD have not been clearly explored, which requires further experiments.

The gut microbiota produces a variety of metabolites that have different roles in blood pressure regulation, and beneficial metabolites include short-chain fatty acids (SCFAs) and vitamins. Short-chain fatty acids are thought to be beneficial for lowering blood pressure primarily through their vascular relaxation and anti-inflammatory effects, in contrast to another metabolite produced by the gut microbiome, trimethylamine N-oxide (TMAO), which is positively associated with high blood pressure (34). TMAO has pro-atherogenic and pro-thrombotic effects, so the gut microbiome produces different metabolites that have different effects on the risk of CHD. Short-chain fatty acids can improve its control, while TMAO is harmful.

The gut microbiota may also influence blood lipids, one of the risk factors for cardiovascular disease. Through metabolites, the broken-chain fatty acids are produced by a variety of gut bacteria, which are important metabolites for protecting against dyslipidemia. The physiological and metabolic activities of the gut microbiota are critical for regulating and maintaining balanced lipid metabolism in humans. *Firmicutes* and *Bacteroides* are the main bacterial groups that affect the changes of blood lipids. Lipid metabolites of the gut microbiota (such as choline, TMAO, and betaine) promote atherosclerosis and increase the risk of cardiovascular disease. Gut microbiota affects the conversion of serum triglycerides and high-density lipoprotein cholesterol. Three mechanisms are likely to cause dyslipidemia. First, the gut flora produces bile salt hydroxylase, which converts bound bile acids into secondary free bile acids. Secondary free bile acids can regulate liver and lipid metabolism through G protein-coupled receptors, and gut microbiota disorders can lead to abnormal bile acid secretion, resulting in dyslipidemia. Second, the gut flora converts choline and carnitine from the host to trimethylamine (TMA), and TMA is converted to TMAO in the liver (35). TMAO can cause dyslipidemia and atherosclerotic plaque by affecting cholesterol transport and metabolism and bile acid levels. Third, SCFAs can inhibit the activity of liver fat synthetase and regulate the distribution of cholesterol in the blood and liver, thus playing a role in reducing serum 3-acylglycerol and cholesterol levels. In addition, patients with dyslipidemia often exhibit high levels of TMAO, which reduces HDL-C levels and hence increases the risk of CHD (36). TMAO can also induce overreaction of platelets and is therefore a risk factor for atherosclerosis. The interaction between TMAO and platelets may promote platelet hyperreactivity and enhance thrombosis *in vivo* by altering platelet-dependent calcium signaling. Platelet hyperreactivity has been reported as a risk factor for cardiovascular events. Recent evidence suggests that TMAO can quickly send signals to cells within minutes. In endothelial or smooth muscle cells, TMAO rapidly induces mitogen-activated protein kinases and NF-κB activation, and causes downstream upregulation of adhesion molecules. Increased TMAO levels were also associated with increased phosphorylation of the SMAD-3 protein. SMAD-3 is a key signal in the transforming growth factor beta (TGF-β) pathway. In animal models, TMAO promotes vascular inflammation and induces aortic endothelial cell activation and upregulation of adhesion proteins. These effects are key mechanisms of acute coronary syndrome. Gut microbiota may also contribute to coronary heart disease through the role of uric acid in serum uric acid level, which may be an independent risk factor for coronary heart disease. Uric acid has oxidizing properties in the body. Elevated blood uric acid levels lead to increased oxygen free radicals in blood uric acid, oxidative stress, vascular endothelial dysfunction, inflammatory responses, and the development of atherosclerosis (37).

In short, intestinal microbiota and its metabolites have a close influence on the risk of coronary heart disease. The disturbance of intestinal microbiota will lead to the disturbance of its metabolites, and then lead to a series of risk factors related to coronary heart disease, such as hypertension, hyperlipidemia, and diabetes. As a result, the combination of systemic and local diseases leads to the occurrence of coronary heart disease.

There are some limitations to our study. First, MR must follow three strict core assumptions, namely, correlation, independence, and exclusion constraints. Nevertheless, we employed a careful study design to avoid any violation of these assumptions. Secondly, the GWAS data of intestinal flora used in our study only reached the genus level, but did not reach the more detailed species level, which also has a certain impact on our study. The impact of specific intestinal bacteria on coronary heart disease needs further experimental verification or more detailed species-level data analysis. Finally, to minimize the effect of racial differences, we only used GWAS data from individuals of European descent for the MR analysis. Therefore, the generalization of our findings to other populations deserves further exploration and verification. In addition, while the MR analysis provides valuable insights into etiology, it is important to note that our findings should be validated through rigorous randomized controlled trials and basic research before being applied to the clinic.

## 5 Conclusion

This study presents multiple benefits. Specifically, it employed MR analysis to establish a causal relationship between gut microbiota and CHD, thereby mitigating the influence of confounding variables and reversing the direction of causality in causal inference. The genetic variation in the gut microbiota was obtained from the most extensive GWAS meta-analysis, ensuring the robustness of the MR analysis. In addition, cross-sectional polymorphisms were identified and eliminated through the utilization of MR-PRESSO and MR-Egger regression intercept term tests. A two-sample MR design with non-overlapping exposure and outcome summary level data was used to avoid bias. We identified nine different species of gut bacteria with potential causal relationship to CHD, but due to the limitations of gut flora database and CHD data, further studies or observational experiments are needed to confirm.

## Data Availability

The original contributions presented in the study are included in the article/**Supplementary Material**, further inquiries can be directed to the corresponding authors.
