# Supplementary material for: Gut microbiota and risk of coronary heart disease: a two-sample Mendelian randomization study
Source: Front Cardiovasc Med. 2024 Mar 25;11:1273666. doi: 10.3389/fcvm.2024.1273666 (PMC10999620; doi:10.3389/fcvm.2024.1273666)

Lentisphaeria

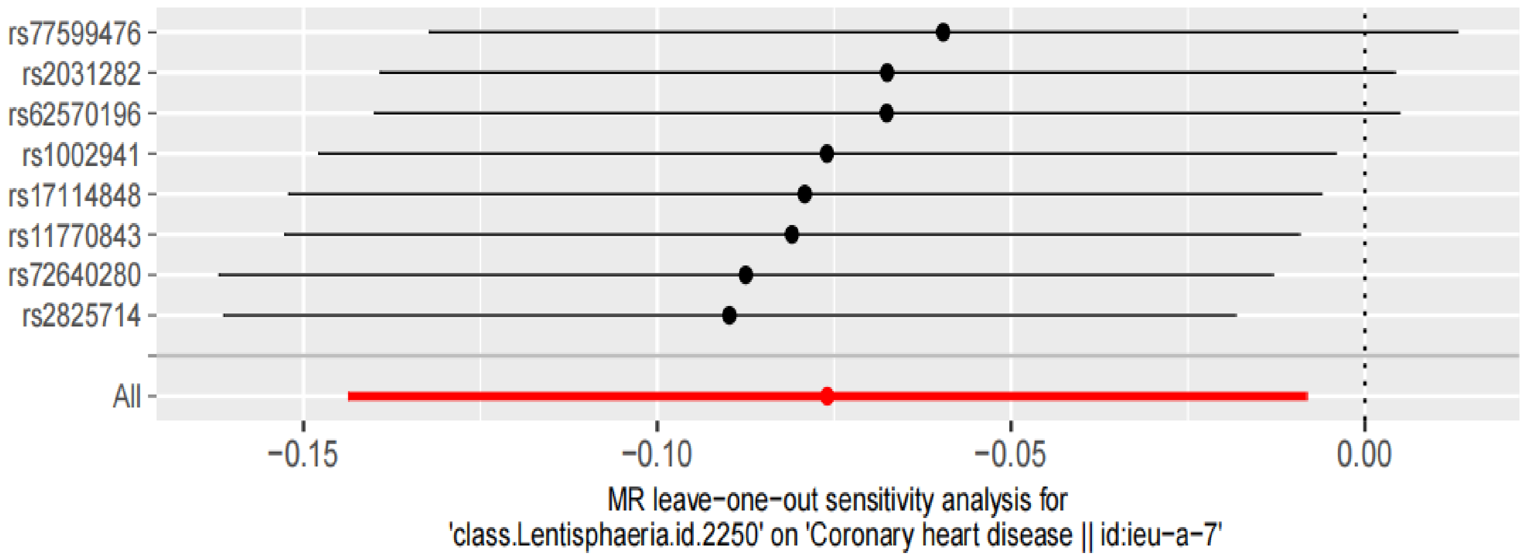

ClostridialesvadinBB60group

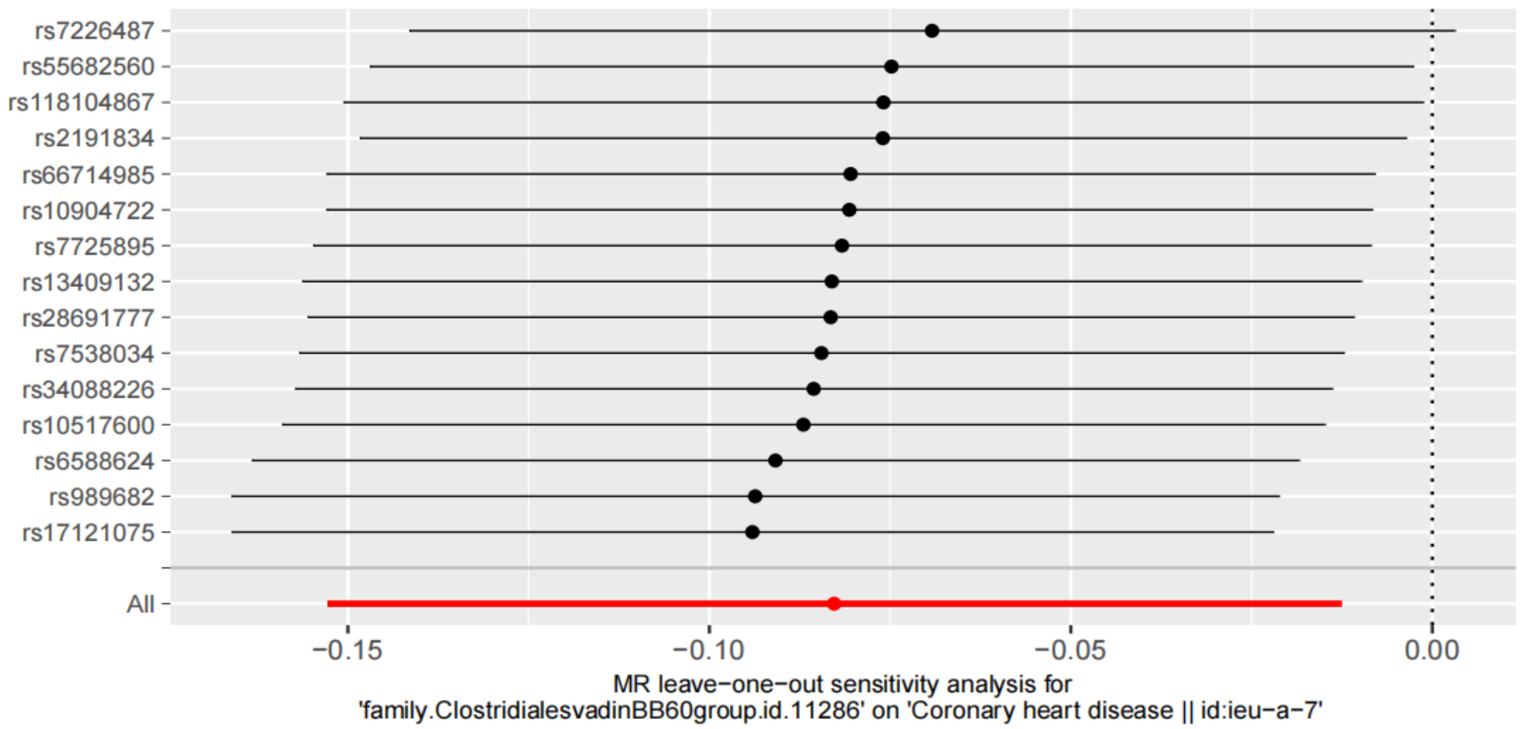

Clostridiuminnocuumgroup

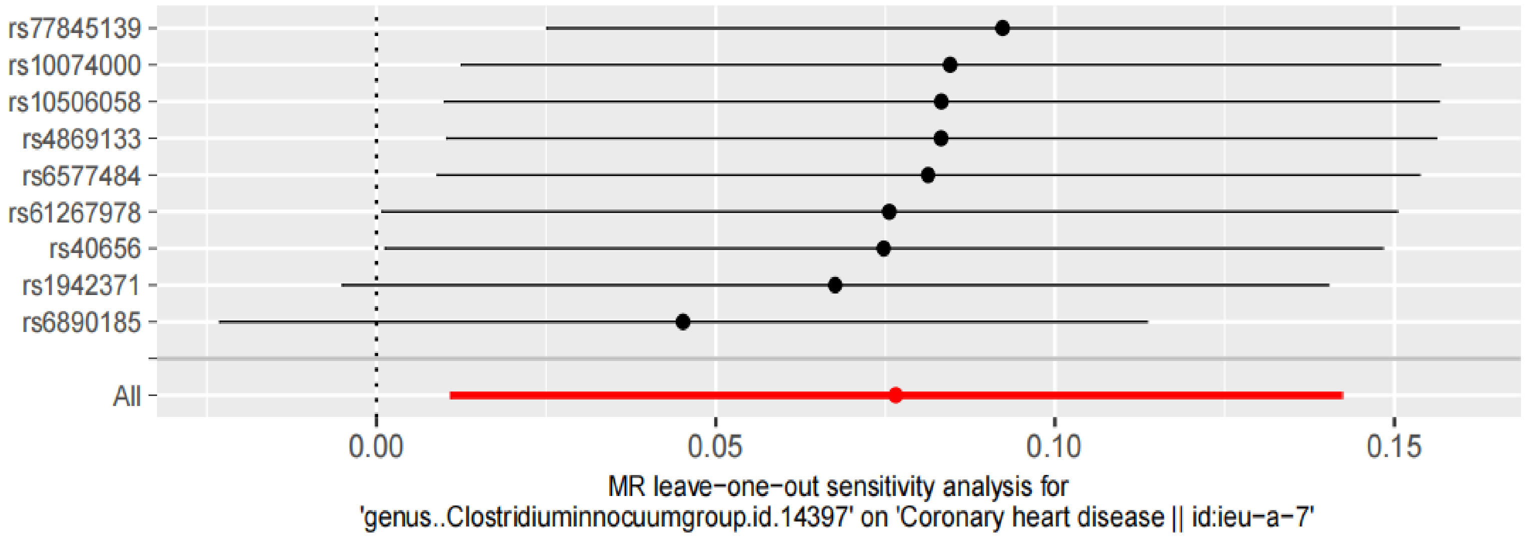

Butyricicoccus

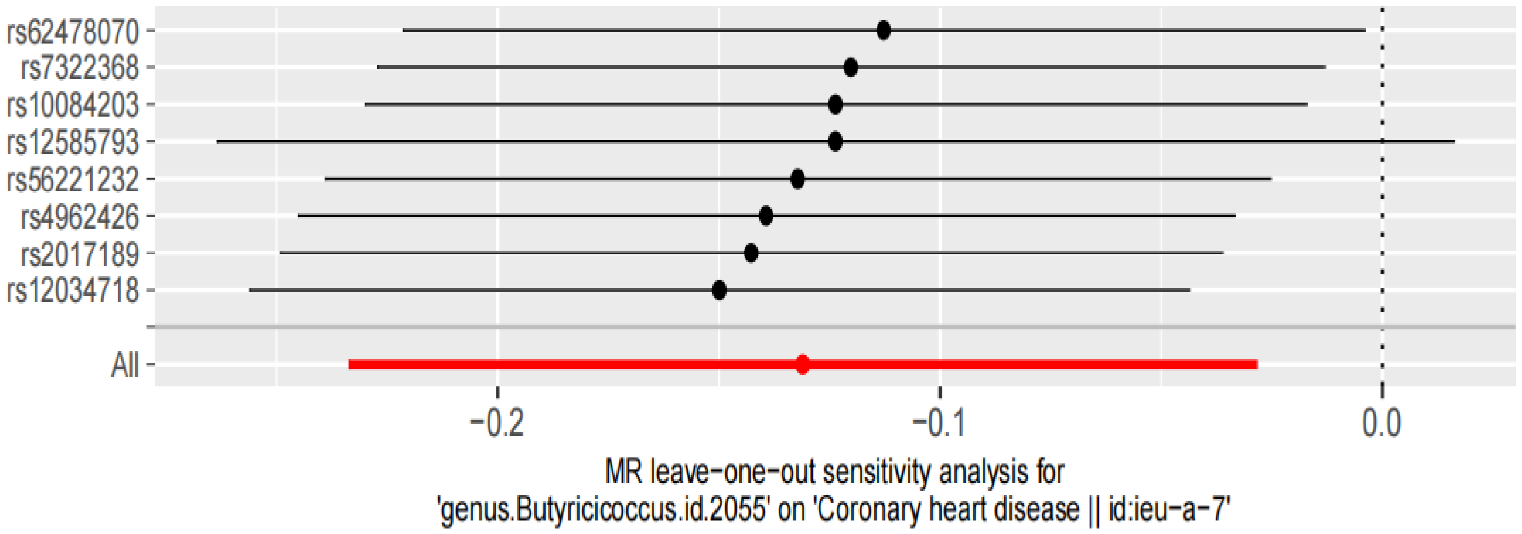

Oxalobacter

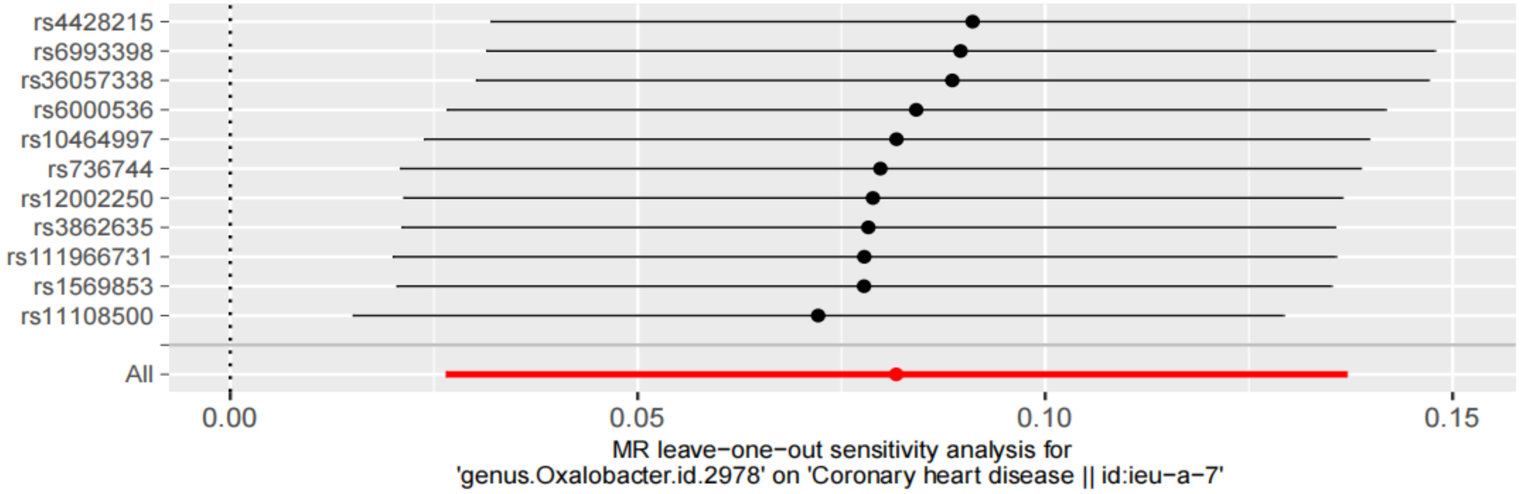

Turicibacter

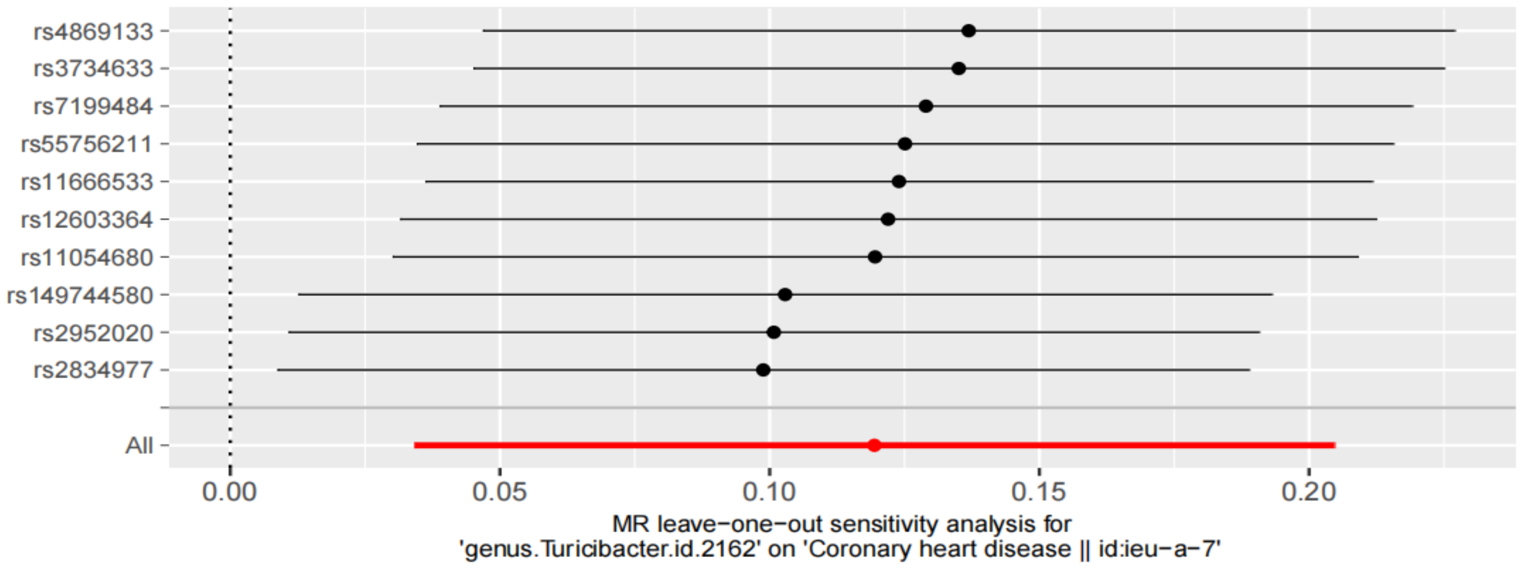

Victivallales

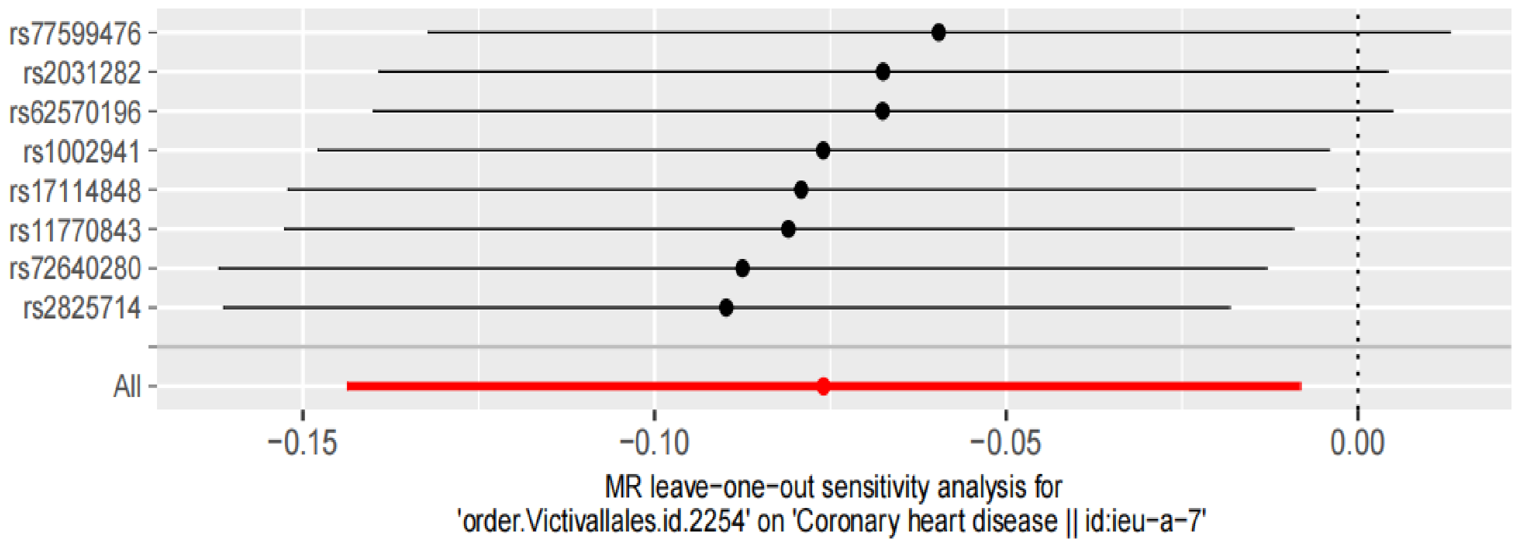

Supplement: Supplementary file 3 [file Datasheet1.pdf]
